# Evaluation of ADMA-DDAH-NOS axis in specific brain areas following nitroglycerin administration: study in an animal model of migraine

**DOI:** 10.1186/s10194-015-0560-2

**Published:** 2015-08-13

**Authors:** Rosaria Greco, Andrea Ferrigno, Chiara Demartini, Annamaria Zanaboni, Antonina Stefania Mangione, Fabio Blandini, Giuseppe Nappi, Mariapia Vairetti, Cristina Tassorelli

**Affiliations:** Laboratory of Neurophysiology of Integrative Autonomic Systems, Headache Science Centre, “C. Mondino” National Neurological Institute, Pavia, Italy; Department of Internal Medicine and Therapeutics, Pharmacology and Toxicology Unit, University of Pavia, Pavia, Italy; Department of Brain and Behavioural Sciences, University of Pavia, Pavia, Italy; Laboratory of Functional Neurochemistry, Center for Research in Neurodegenerative Diseases, “C. Mondino” National Neurological Institute, Pavia, Italy

**Keywords:** Nitroglycerin, Migraine, Rat brain, nNOS, eNOS, ADMA, DDAH

## Abstract

**Background:**

Nitric oxide (NO) is known to play a key role in migraine pathogenesis, but modulation of NO synthesis has failed so far to show efficacy in migraine treatment. Asymmetric dimethylarginine (ADMA) is a NO synthase (NOS) inhibitor, whose levels are regulated by dimethylarginine dimethylaminohydrolase (DDAH). Systemic administration of nitroglycerin (or glyceryl trinitrate, GTN) is a NO donor that consistently induces spontaneous-like headache attacks in migraneurs. GTN administration induces an increase in neuronal NOS (nNOS) that is simultaneous with a hyperalgesic condition. GTN administration has been used for years as an experimental animal model of migraine. In order to gain further insights in the precise mechanisms involved in the relationships between NO synthesis and migraine, we analyzed changes induced by GTN administration in ADMA levels, DDHA-1 mRNA expression and the expression of neuronal and endothelial NOS (nNOS and eNOS) in the brain. We also evaluated ADMA levels in the serum.

**Methods:**

Male Sprague–Dawley rats were injected with GTN (10 mg/kg, i.p.) or vehicle and sacrificed 4 h later. Brain areas known to be activated by GTN administration were dissected out and utilized for the evaluation of nNOS and eNOS expression by means of western blotting. Cerebral and serum ADMA levels were measured by means of ELISA immunoassay. Cerebral DDAH-1 mRNA expression was measured by means of RT-PCR. Comparisons between experimental groups were performed using the Mann Whitney test.

**Results:**

ADMA levels and nNOS expression increased in the hypothalamus and medulla following GTN administration. Conversely, a significant decrease in DDAH-1 mRNA expression was observed in the same areas. By contrast, no significant change was reported in eNOS expression. GTN administration did not induce any significant change in serum levels of ADMA.

**Conclusion:**

The present data suggest that ADMA accumulates in the brain after GTN administration *via* the inhibition of DDAH-1. This latter may represent a compensatory response to the excessive local availability of NO, released directly by GTN or synthetized by nNOS. These findings prompt an additional mediator (ADMA) in the modulation of NO axis following GTN administration and offer new insights in the pathophysiology of migraine.

## Background

Nitric oxide (NO) may function as a signaling molecule in controlling neuronal activity and plays an important role in governing sensory inputs during migraine [1]. Endogenous NO is produced by the constitutive isoforms of NO synthase, endothelial nitric oxide synthase (eNOS) and neuronal nitric oxide synthase (nNOS). Asymmetric dimethylarginine (ADMA), a major endogenous inhibitor of NOS, inhibits NO production *in vivo* and *in vitro* [2, 3]. Besides ADMA, two other forms of methylated arginine — which can be considered arginine analogues — have been identified in eukaryotes: *NG*-monomethyl-l-arginine (l-NMMA), and ω-*NG*,*N*′*G*-symmetric dimethylarginine (SDMA) [4]. All three methylated arginines (ADMA, l-NMMA and SDMA) are inhibitors of arginine transport at superphysiological concentrations, while the physiological relevance of this inhibition remains unclear [5, 6]. Circulating ADMA is present at higher concentrations than l-NMMA and is often considered to be the principal inhibitor of NOS activity [2]. Most of ADMA is degraded by dimethylarginine dimethylaminohydrolase (DDAH), which hydrolyzes ADMA to L-citrulline and dimethylamine [7]. Therefore, this enzymatic pathway is a potential endogenous mechanism for the regulation of NO production by competitive inhibition. ADMA has been associated to cardiovascular risk [7, 8] as it seems involved in the development and progression of cardiovascular disease, *via* the inhibition of eNOS activity and increased production of superoxides [9]. However, high levels of ADMA and increased DDAH-1 expression have been detected in the brain, and spinal cord, thus suggesting a possible role for the ADMA-DDAH pathway in the modulation of neuronal activity [10–12]. This hypothesis seems even more compelling when considering that DDAH-1 co-localizes with nNOS [11]. Increased ADMA levels seem to induce endothelial dysfunction and oxidative stress [9, 12], two potential factors involved in migraine pathogenesis [13, 14]. Available data on ADMA plasma levels and migraine have yielded inconclusive findings so far [15–17] and there is no information on ADMA/DDAH pathway in animal models of migraine.

Exogenous NO, released by nitroglycerin (or glyceryl trinitrate, GTN), induces migraine-like headache in predisposed subjects and it has been used as a human [18, 19] and animal model for the study of migraine [20–22]. GTN also activates the NO synthetic pathway in humans and rats [23, 24].

In order to gain new insights in ADMA-DDAH-NO axis in migraine pain, in this study we investigated changes in brain and serum ADMA levels, together with nNOS and eNOS expression and DDHA-1 expression in discrete areas of the rat brain following GTN administration.

## Methods

Male Sprague–Dawley rats were injected with GTN (10 mg/kg, i.p.) or vehicle and sacrificed 4 h after the injection. The principles of the Helsinki declaration and IASP’s guidelines for pain research in animal were rigorously applied [25]. Animals were housed in plastic boxes in groups of 2 with water and food available *ad libitum* and kept on a 12:12 h light–dark cycle. A total of 28 animals were used for the experiments and all procedures were in accordance with the European Convention for Care and Use of Laboratory Animals and were approved by the local animal ethic committee of the University of Pavia (Document n. 2, 2012). GTN [Bioindustria L.I.M. Novi Ligure (AL), Italy] was prepared from a stock solution of 5.0 mg/1.5 mL dissolved in 27 % alcohol and 73 % propylene glycol. For the injections, GTN was further diluted in saline (0.9 % NaCl) to reach the final concentration of propylene glycol (PG) 16 % and alcohol 6 % and administered at a dose of 10 mg/kg. A solution of saline (0.9 % NaCl), PG 16 % and alcohol 6 % was used as vehicle (CT group).

On the basis of the distribution of the nuclei that are known to be activated by GTN and involved in migraine pain, the following discrete brain areas were dissected out 4 h after GTN or vehicle administration and used for analysis: medulla-pons, containing nucleus trigeminalis caudalis (NTC), nucleus tractus solitarius and area postrema; mesencephalon, containing ventrolateral column of the periaqueductal grey and parabrachial nucleus, and hypothalamus, containing the paraventricular and supraoptic nuclei of the hypothalamus.

### Western blotting

Rats (*N* = 6 per experimental group) were perfused transcardially with 250 ml cold saline, 4 h after GTN or vehicle administration. Brains were immediately removed and chopped into parts; brain areas of interest were dissected out and used for the preparation of total extracts. The samples were homogenized on ice with a homogenizer in at least 5 volumes of modified RIPA buffer (Tris 50 mM, pH 7.4, NaCl 150 mM, EDTA 1 mM, SDS 0,2 %) supplemented with cocktail inhibitors protease. Then, they were incubated on ice for 20 min. The tissue lysate was centrifuged at 10,000 × *g* for 45 min at 4 °C and supernatants stored at −80 °C. Protein assay was performed by bicinchoninic acid (BCA) method. A 20 μg of protein were submitted to SDS-poliacrylamide gels 10 % and transferred onto a PVDF membrane (Amersham Biosciences). After blocking with 5 % dry milk, the blots were probed overnight at 4 C° with rabbit polyclonal anti-nNOS serum (1:1000; Cayman Chemical) or anti-eNOS serum (1:1000; Santa Cruz Bioctenology) and then probed for 1 h with an anti-rabbit horseradish peroxidase coupled secondary antibody (1:10000; Amersham Biosciences). An enhanced chemiluminescence system (ECL Advance; Amersham Biosciences) was used for visualization. Membranes were also probed with a rabbit polyclonal anti-β actin antibody (1:1000; Santa Cruz Biotechnology) as a housekeeping protein.

For semiquantitative analysis, a Bio-Rad GS800 densitometer was used. NOS expression was evaluated in each sample by dividing the optical density of the NOS band by the intensity of the optical density of the band corresponding to the housekeeping protein*.* The specificity of the antibodies was confirmed by immunoprecipitation with a specific blocking peptide.

### Enzyme-linked immunosorbant assays (ELISA)

Rats (*N* = 8 per experimental group) were injected with GTN (10 mg/kg i.p.) or vehicle and then killed with a lethal dose of anaesthetic 4 h after treatment. Their brains were immediately chopped into parts; brain areas of interest were dissected out and frozen at −80 °C until further processing.

Blood was drawn from the vena cava and centrifuged at 3000 g for 10 min at 4 °C.

ADMA levels (ng/mg proteins or nmol/ml) were quantified by ELISA kit (Antibodies Online) according to the manufacturer’s instructions.

### Real-time polymerase chain reaction

Rats (*N* = 6 per experimental group) were injected with GTN (10 mg/kg i.p.) or vehicle and then killed with a lethal dose of anaesthetic 4 h after treatment. Their brains were immediately chopped into parts and frozen at −80 °C until further processing.

DDAH-1 mRNA expression was analyzed by a real-time polymerase chain reaction (RT-PCR) and total RNA was isolated from the cerebral samples with Trizol reagent in accordance with the method of Chomczynski and Mackey [26]. RNA was quantified by measuring the absorbance at 260/280 nm. cDNA was generated using the iScript cDNA Synthesis kit (Bio-Rad) following the supplier's instructions. Gene expression was analyzed using the Fast Eva Green supermix (Bio-Rad). As regards housekeeping, gene glyceraldehyde 3-phosphate dehydrogenase (GAPDH) was used. The expression of the housekeeping gene remained constant in all the experimental groups considered. The amplification was performed through two-step cycling (95–60 °C) for 45 cycles in a light Cycler 480 Instrument RT-PCR Detection System (Roche) following the supplier's instructions. All samples were assayed in triplicate. Gene expression was calculated using the ΔCt method.

### Statistical evaluation

Data are expressed as mean ± SD. Comparisons between groups (GTN and CT) were performed using the Mann Whitney test. The minimum level of statistical significance was set at *p* < 0.05.

## Results

### nNOS and eNOS expression

**Fig. 1:**
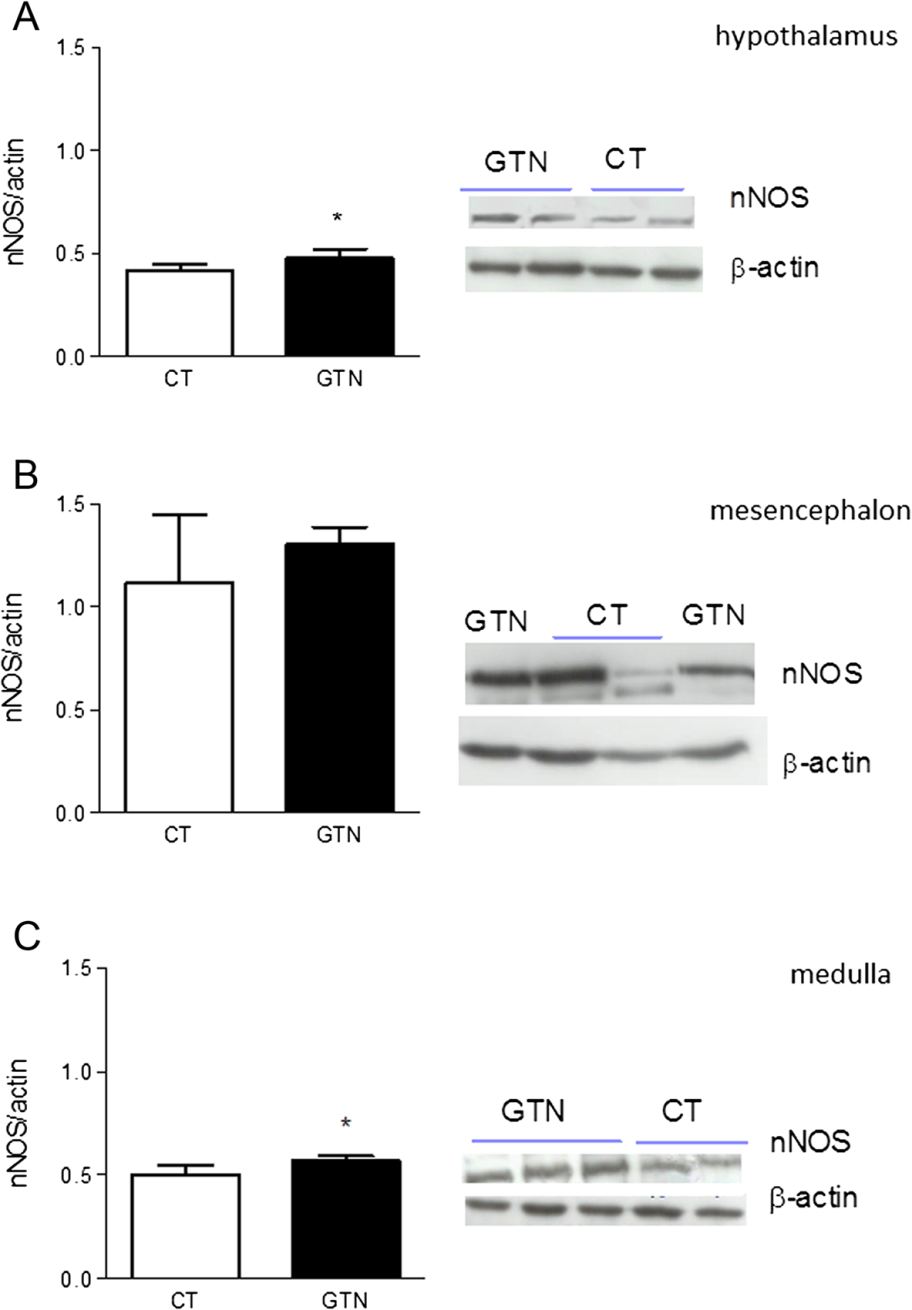
nNOS expression in homogenates of hypothalamus (**a**), mesencephalon (**b**) and medulla (**c**) of rats injected with glyceryl trinitrate (GTN) or vehicle (CT). The histograms illustrate the densitometric analysis representing expression levels of nNOS (155KDa), evaluated as the ratio vs β-actin (39 kDa). The latter protein was used as a housekeeping protein on the same membrane previously incubated with nNOS. nNOS expression was evaluated after 4 h of GTN or vehicle injection. In the right of each panel are illustrated representative western blots of nNOS protein. Data are expressed as mean ± SD. Mann Whitney test, *p < 0.05 vs vehicle (CT)

**Fig. 2:**
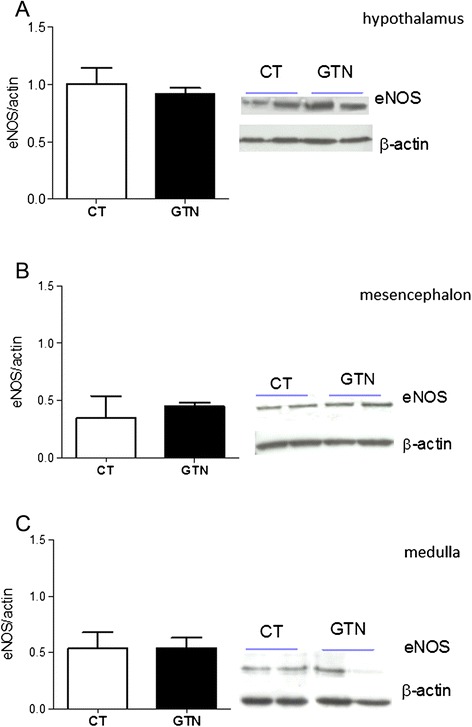
eNOS expression in homogenates of hypothalamus (**a**), mesencephalon (**b**) and medulla (**c**) of rats injected with glyceryl trinitrate (GTN) or vehicle (CT). The histograms illustrate the densitometric analysis representing expression levels of eNOS (130KDa) as ratio vs β-actin (39 kDa). The latter protein was used as a housekeeping protein on the same membrane previously incubated with eNOS. In the right of each panel are illustrated representative western blots of eNOS protein. eNOS expression was evaluated after 4 h of GTN or vehicle injection. Data are expressed as mean ± SD. Mann Whitney test, *p < 0.05 vs vehicle (CT)

Western blotting analyses using the anti-nNOS antibody revealed the presence of one band at 155 KDa. In the GTN Group, the intensity of this band was significantly increased in the hypothalamus and medulla, when compared to the control group (Fig. 1). By contrast, no change in eNOS expression (135KDa) was detected in any of the cerebral areas under evaluation after GTN administration (Fig. 2).

### AMDA levels

**Fig. 3:**
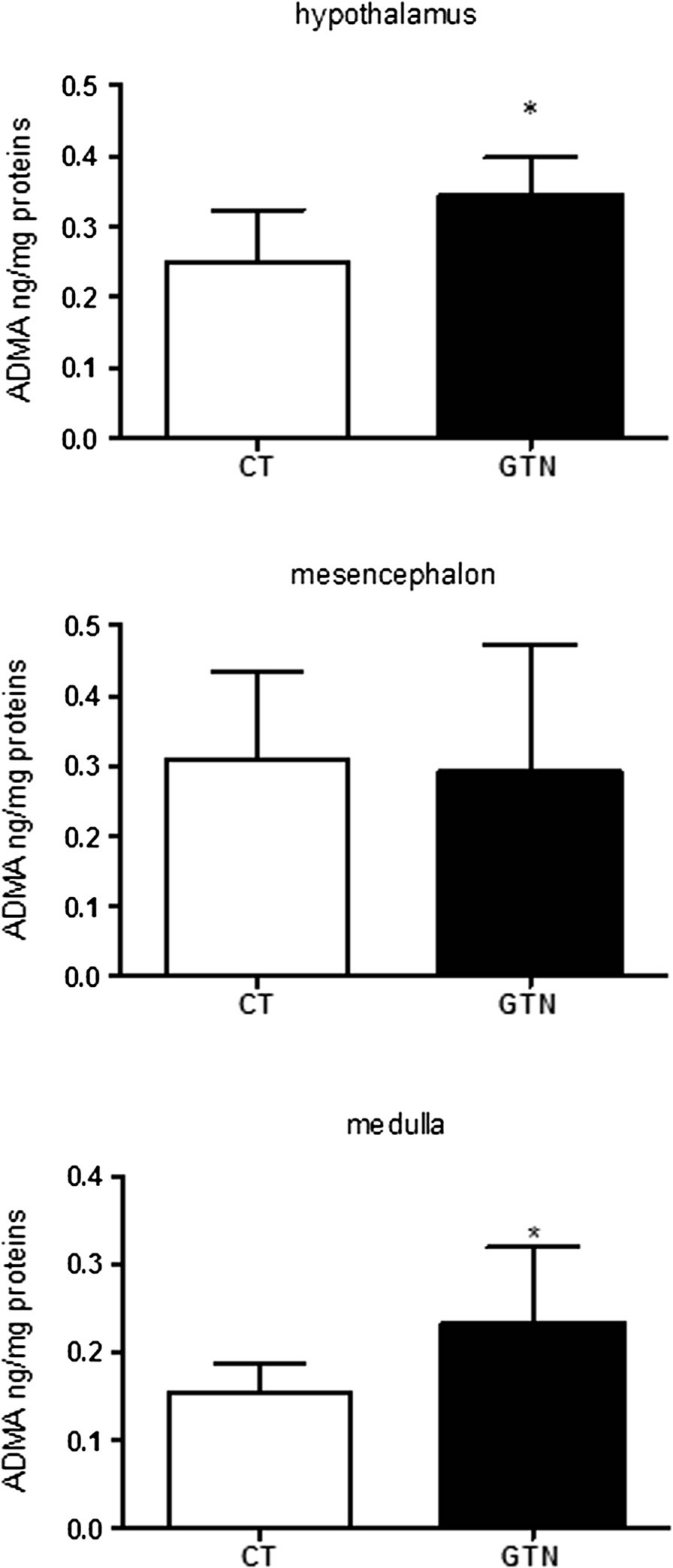
Cerebral levels of ADMA in rats injected with glyceryl trinitrate (GTN) or vehicle (CT). ADMA levels were evaluated after 4 h of GTN or vehicle injection. Data are expressed as mean ± SD, Mann Whitney test, *p <0.05 vs vehicle (CT)

**Fig. 4:**
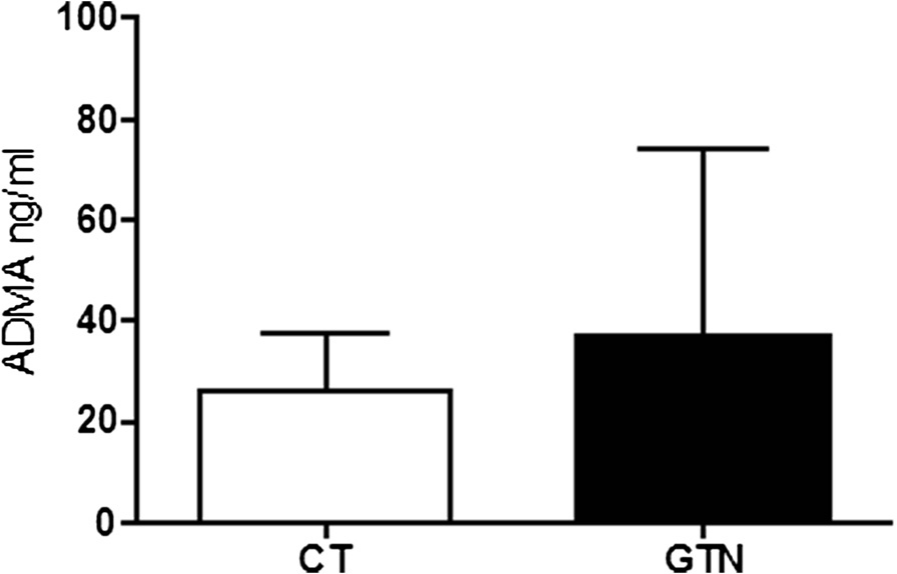
Serum levels of ADMA in rats injected with glyceryl trinitrate (GTN) or vehicle (CT). ADMA levels in serum were evaluated after 4 h of GTN or vehicle injection. Data are expressed as mean ± SD

ADMA levels were significantly increased in the hypothalamus and medulla of GTN treated rats, when compared to CT group. Conversely, we did not detect any significant differences in mesencephalon (Fig. 3). No significant difference was observed in serum ADMA concentrations between GTN and CT groups (Fig. 4).

### DDAH-1mRNA expression

**Fig. 5:**
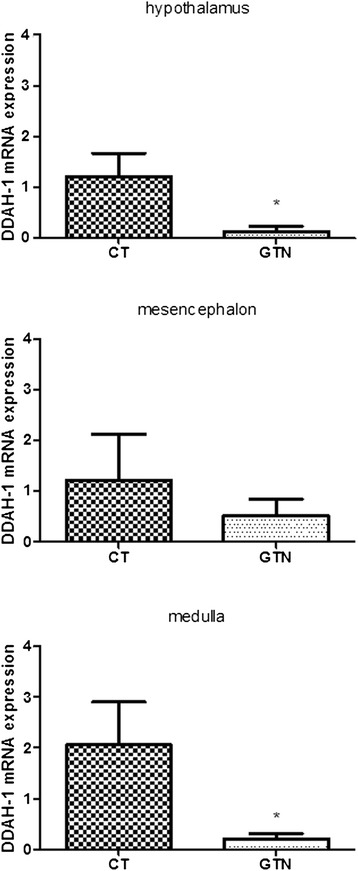
DDAH-1 mRNA expression in specific brain areas, 4 h after glyceryl trinitrate (GTN) or vehicle (CT) administration. Data are expressed as mean ± SD, Mann Whitney test, *p <0.05 vs vehicle (CT)

DDAH-1 mRNA expression was significantly decreased in the hypothalamus and in the medulla of rats treated with GTN when compared to CT group. No significant difference in DDAH-1 mRNA expression was found in the mesencephalon of rats treated with GTN when compared to CT group (Fig. 5).

## Discussion

Strong evidence supports the idea that NO plays a pivotal role in the pathogenesis of migraine [27, 28], a disorder characterized by pain sensitization associated with cranial vascular changes [29–31], but mechanisms and modalities of NO activity are still largely unknown. Systemic GTN activates neuronal groups in selected areas of the rat brain involved in nociception [21, 32, 33] and induces spontaneous-like attacks in migraineurs *via* multimodal mechanisms that include GTN- induced vasodilation, peripheral sensitization induced by the increased availability of NO at the trigeminovascular level, and possibly also central sensitization [34–37].

GTN administration induces an increase in nNOS that is simultaneous with a hyperalgesic condition and neuronal activation in brain areas involved in migraine pain [38, 39], thus suggesting that NOS inhibition may be a potential therapeutic target for migraine. Experimental and clinical studies suggest that NOS inhibition influences the activation of the trigeminal vascular system and that nonselective NOS inhibition is associated to antimigraine activity [40, 41]. Clinical application of non selelctive NOS inhibition is however hindered by the cardiovascular effects, *i.e.,* increase of mean arterial pressure and a decrease of heart rate for its pharmacokinetic profile [41].

ADMA, is a methylated arginine found in plasma, urine and different tissues [2], which is released when methylated proteins are degraded into their amino acid components during hydrolytic protein turnover [8]. ADMA blocks NO synthesis and can induce endothelial dysfunction, both *in vivo* and *in vitro* [2, 3], and cause oxidative stress [42], two potential factors involved in migraine pathogenesis [13, 14]. DDAH regulates ADMA levels and NO signalling *in vivo* and ADMA/DDAH system is considered as a novel pathway for modulating NO production [43]. DDAH-1 predominates in tissues that express nNOS, whereas DDAH-2 predominates in tissues expressing eNOS [44]. Since large amounts of ADMA and DDAH-1 have been detected in the brain and spinal cord, probably ADMA/DDAH-1 pathway may have a role also in neuronal, inflammatory and other non-cardiovascular pathologies, as migraine pain, where NO has pivotal role [15]. Uzar *et al.*, [15] found elevated plasma levels of ADMA and NO in migraine patients as compared to control subjects, suggesting that an increase in ADMA levels in migraine might represent a compensatory mechanism for blocking NO production and NO-induced excessive vasodilatation [15]. However, differences in ADMA and NO levels when comparing ictal and interictal levels in migraineurs yielded inconclusive findings [15–17]. To the best of our knowledge, no information is available on cerebral ADMA and DDAH-1 expression in experimental animal models of migraine.

In this study, we evaluated the simultaneous changes in ADMA levels and DDAH-1 mRNA expression in brain areas in an animal model specific for migraine in order to evaluate whether ADMA-DDAH-pathway may be involved in migraine. We also evaluated nNOS and eNOS expression in the same brain areas, and ADMA levels in the venous blood, drawn from the vena cava. Our findings show that AMDA levels significantly increased in the hypothalamus and medulla 4 h after GTN administration, the timing where we observe neuronal activation and hyperlagesia. This increase was associated to the inhibition of DDAH-1 expression and to the increase in nNOS expression in the same areas. eNOS expression instead was not affected. Taken together, these results suggest that the increase in brain NO availability, secondary to GTN exposure [45], may have interfered with DDAH-1 expression, possibly *via* S-nitrosylation of DDAH-1 active site [46, 47]. Indeed, deletion of DDAH-1 gene, or the inhibition of its transcription, is associated with an increase of ADMA levels [48]. Alternatively, DDAH-1 expression may have been inhibited *via* GTN-induced oxidative stress [49] or GTN-induced activation of inflammatory pathway [50, 51]. Previous reports have indeed shown that DDAH activity and protein expression may be markedly reduced during oxidative stress and/or inflammation [52–54].

Circulating levels of ADMA were not affected by GTN treatment to suggest that GTN interferes with DDAH-1 expression only at cerebral level, but not at peripheral level such as the liver, where high net hepatic uptake of ADMA occurs [55]. In agreement with a selective ‘neuronal’ activity of AMDA in this experimental paradigm is the absence of changes observed in eNOS.

## Conclusions

The present data suggest that ADMA accumulates in the brain after GTN administration *via* the inhibition of DDAH-1. This latter may represent a compensatory response to the excessive local availability of NO, released directly by GTN or synthetized by nNOS. These findings prompt an additional mediator (ADMA) in the modulation of NO axis following GTN administration and offer new insights in the pathophysiology of migraine.

## Abbreviations

GTN: Nitroglycerin or Glyceryl trinitrate
ADMA: Asymmetric dimethylarginine
NO: Nitric oxide, nNOS, neuronal nitric oxide synthase
eNOS: endothelial nitric oxide synthase
NMMA: NG-monomethyl-l-arginine (l-)
SDMA: ω NG,N′G-symmetric dimethylarginine
DDAH: dimethylarginine dimethylaminohydrolase
